# Comparative evaluation of tissue adhesives and conventional sutures in periodontal flap surgery: A randomized clinical study

**DOI:** 10.6026/973206300220241

**Published:** 2026-01-31

**Authors:** Ankita Garg, Preksha Agrawal, Geeta Tripathi, Gaurav Jain

**Affiliations:** 1 Department of Dentistry, Shyam Shah Medical College, Rewa, Madhya Pradesh, India; 2 Department of Obstetrics and Gynaecology, Shri Ramkrishna Institute of Medical Sciences and Sanaka Hospitals, Durgapur, West Bengal, India; 3 Department of Pulmonology and Sleep Medicine, Batra Hospital and Medical Research Centre, New Delhi, India

**Keywords:** Periodontal surgery, tissue adhesive, cyanoacrylate, fibrin sealant, sutures, wound healing, tisseel, amcrylate

## Abstract

Post-surgical discomfort, delayed soft tissue healing and risk of infection remain significant challenges associated with conventional suturing methods in periodontal flap surgeries. Therefore, it is of interest to evaluate four different groups (n=10 each); Group 1 - silk sutures, Group 2 - vicryl sutures, Group 3 - fibrin sealant (Tisseel) and Group 4 - isoepoxy 2-cyanoacrylate adhesives (Amcrylate) and compared effects on wound healing, postoperative discomfort and clinical outcomes in periodontitis patients. Phase I therapy was followed by full-thickness mucoperiosteal flaps elevation, debridement and closure conducted as specified. The measurement of healing was done at 1, 2, 4 and 12 weeks by the use of Landry Healing Index, Visual Analog Scale (VAS) of pain, Plaque and Gingival Indices, Probing Pocket Depth (PPD) and Clinical Attachment Level (CAL). Tissue glues, especially fibrin-based sealants, provided periodontal flap closure with clinically reliable, biocompatible and patient-friendly option which enhances faster hemostatic and early healing.

## Background:

Periodontitis is a persistent inflammatory illness of the tissue that supports the teeth and results in loss of progressively more and more of attachment and alveolar bone resorption [1]. Periodontal therapy should eventually aim at the restoration of health and functioning by restoring the integrity of the attachment apparatus [2]. Flap surgery continues to be a conclusive form of managing severe periodontitis that can be neither removed by the nonsurgical mode of treatment of deep periodontitis. Proper wound approximation and an infection free environment of healing requires success [3]. Historically, flap closure has been the gold standard of using sutures. Among other things, non-resolvable silk sutures are preferred due to their facilitation of ease of use and knot strength. Nevertheless, they can breed bacteria, cause tissue inflammation and need a follow-up appointment to remove them [4]. Sutures made of polyglactin 910 (vicryl) will decrease the suture removal process and might result in premature removal, tissue response, or delayed healing when left behind [5]. Intense focus has therefore shifted towards tissue adhesives which might somehow be used to address these limitations. Tissue adhesives offer instantaneous hemostasis, no needle-punctures and they seal the wound without mechanical injuries [6]. These are classified into two, namely, natural polymer-based sealants (e.g., fibrin, albumin, gelatin) and synthetic adhesives (cyanoacrylates) [7]. Sealants as fibrin such as Tisseel replicate physiological coagulation -thrombin converts fibrinogen to fibrin creating a permanent clot and this clot merges with host tissue [8]. Synthetic cyanoacrylate derivatives, including Amcrylate, are exothermic polymerized in a wet environment, to create a strong and biocompatible bond providing bacterial resistance and lower chair time [9]. Initial results have demonstrated encouraging results of fibrin and cyanoacrylate sealants in oral and maxillofacial surgery. Prato *et al.* proved that fibrin glue is safer and more stable than sutures that favor the stability of flaps and decrease psychological pain after surgical operations [10]. On the same note, cyanoacrylate fixed bonding agents have demonstrated fast response to polymerization, reasonable tensile strength and low inflammatory nature [11]. Therefore, it is of interest to compare tissue adhesives and conventional sutures for periodontal flap closure in a randomized clinical setting to evaluate their effects on wound healing, postoperative discomfort and clinical outcomes in periodontitis patients.

## Materials and Methods:

## Study design and population:

A randomized, four-arm, split-mouth clinical study, conducted at Department of Periodontics, K. D. Dental College and Hospital, Mathura, UP, India, involving 40 sites from 10 systemically healthy patients (age 25-50 years) diagnosed with moderate-to-severe chronic periodontitis. Ethical approval was obtained from the institutional review board and informed consent was secured from all participants in accordance with the Helsinki Declaration (2013).

## Inclusion criteria:

[1] Clinical attachment loss ≥4 mm and probing depth ≥5 mm in at least four quadrants.

[2] Good oral hygiene compliance post Phase I therapy.

## Exclusion criteria:

Smokers, pregnant/lactating women, systemic conditions affecting healing (e.g., diabetes, coagulation disorders) or recent antibiotic use.

## Surgical procedure:

After Phase I therapy (scaling and root planing), selected sites were randomly allocated as follows:

[1] Group 1: Silk sutures (Ethicon 3-0 braided silk).

[2] Group 2: Vicryl sutures (Ethicon 3-0 polyglactin 910).

[3] Group 3: Fibrin sealant (Tisseel, Baxter).

[4] Group 4: Cyanoacrylate adhesive (Amcrylate, isoamyl 2-cyanoacrylate).

Following local anesthesia (2% lignocaine with 1:80,000 adrenaline), full-thickness mucoperiosteal flaps were raised by intracrevicular incisions.

Granulation tissue was removed, roots were planed and the area irrigated with sterile saline.

[1] In suture groups, flaps were approximated with interrupted sutures.

[2] In adhesive groups, the flaps were positioned and thin layers of the respective sealant applied to the margins under gentle pressure for 60 seconds.

## Postoperative protocol:

All patients received amoxicillin 500 mg TDS and ibuprofen 400 mg TDS for 3 days and were instructed to rinse twice daily with 0.12% chlorhexidine for two weeks. Sutures were removed after 7 days. Patients were evaluated at 1, 2, 4 and 12 weeks postoperatively.

## Clinical parameters evaluated:

[1] Landry Healing Index

[2] Visual Analog Scale (VAS) for pain

[3] Plaque Index (Silness & Löe)

[4] Gingival Index (Löe & Silness)

[5] Probing Pocket Depth (PPD)

[6] Clinical Attachment Level (CAL)

All measurements were performed by a single calibrated examiner using a UNC-15 probe and an acrylic stent for reproducibility.

## Statistical analysis:

The data were performed using SPSS v25.0. Intra and intergroup comparison of nonparametric Friedman and Kruskal Wallis were used respectively. The p-value of less than 0.05 was regarded as statistically significant.

## Results:

**Figure 1:**
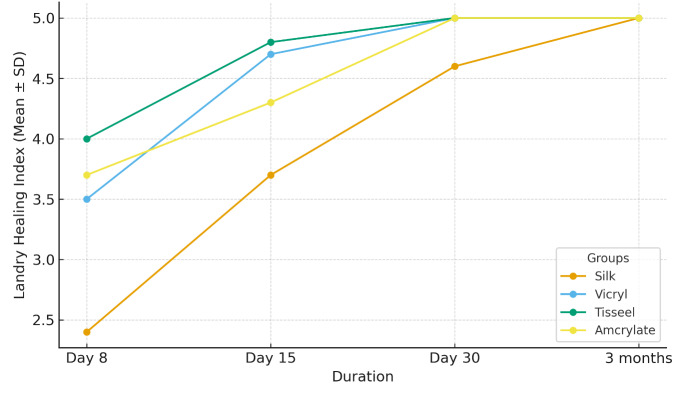
Landry healing index comparison among groups over time

**Figure 2:**
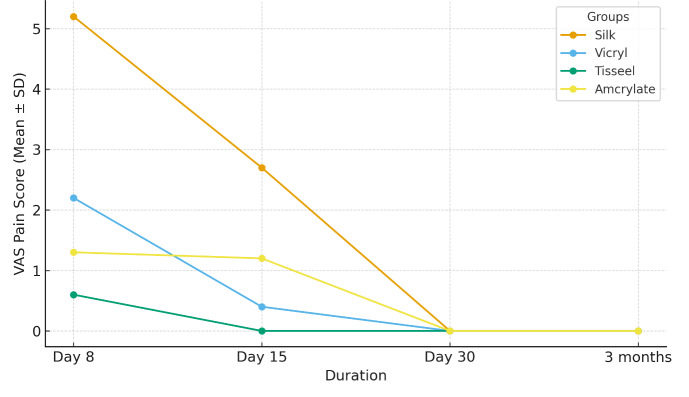
Vas pain score trends among groups over time

No adverse allergic reactions and wound dehiscence were recorded among all the patients that underwent the procedures. A significant reduction in the time required by the operative was found in adhesive groups which exhibited improved initial hemostasy. Tisseel and Amcrylate groups did not produce as much postoperative discomfort as sutures did. Mean Landry Healing Index in the silk suture group (2.40+0.52) was lowest at day 8 and Tisseel group (4.00+0.67) the highest. By day 15 improvements were made by all groups with Tisseel and Vicryl showing quite close healing after one month (p<0.05) (Table 1). Tissue adhesive groups showed less VAS scores during the study. On day 15, the mean VAS of Tisseel was 0.0±0.0 and Amcrylate was 1.2 0.42 and silk was unchanged at 2.7 0.95 (p<0.001) (Table 2). At day 8, silk sites showed higher plaque and gingival scores. By one month, all groups exhibited negligible values (p>0.05) (Table 3). Favored reduction in PPD and CAL were noted in all groups after 3 months. There were no statistically significant differences between the intergroups (p>0.05) (Table 4). The overall mean landry healing index and the VAS pain scores over time for all the groups is visualized in Figure 1 and Figure 2 respectively.

## Discussion:

The use of tissue adhesives as alternatives to traditional methods of suturing during periodontal flap surgery is restated. It was found that fibrin and cyanoacrylate groups had better early wound healing, reduced postoperative discomfort and similar long-term periodontal stability as compared to silk and Vicryl suture [12]. This is due to its biomimetic action making the fibrin sealant to have a better early healing. The terminal phases of the coagulation cascade are recapitulated with fibrin adhesives producing a stable fibrin framework which facilitates fibroblast migration, angiogenesis and epithelial regeneration [13]. Not only is hemostasis guaranteed by this matrix, but also, offers the tissue repair with a biologically compatible scaffold. The reason is that on the sites treated with fibrin, wound clots were stabilized much faster, which indicated that the values of Landry Healing Index on day 8 and day 15 were much higher than in the sutured sites (p=0.00). Moreover, the reduced inflammatory reaction and the lack of injuries in the case of the stitches corrected the comfort of the patient in the postoperative period. Acylate adhesives like Amcrylate gave a good result, the healing and VAS scores were near to those of fibrin. Cyanoacrylates are used as a polymer that form a structurally tough bond and close the flap margins when subjected to moisture [14]. Their bacteriostatic nature, as well as ability to be broken down by enzymes, helps them to have lower rates of infection as well as hemostasis [15]. The polymerization exothermic effect and increased rigidity over fibrin matrices may however explain their slight low scores in early healing. Nevertheless, after the initial month of post-surgery, the clinical results of Amcrylate and Tisseel healing were the same (p=0.01). Traditional sutures, in particular, braided silk had the most substantial low scores of early healing and highest discomfort. Silk sutures are also known to have a big reaction on the tissue as a result of capillarity and retention of plaque [16]. Vicryl sutures were better because they were resorbable and their texture was smoother yet they elicited mild tissue irritation. The trends in the VAS reduction factors as noted in this study are consistent with those of past research that indicates that lack of puncture trauma as well as suture removal are key factors that considerably reduces the postoperative pain and anxiety [17]. Although, there was an increase in early healing, PPD and CAL at 3 months showed no statistically significant change (p=0.56 and p=0.10, respectively). This implies that, although adhesives improve the immediate healing process, the long-term regenerative process is controlled more by the underlying surgical debridement and patient adhesion and not the surgical material [18]. The clinical benefits of adhesives are not limited to healing: the shortened chairside time, the avoidance of suture removal visits and decreased risk of needlestick injuries is beneficial both to the operator and the patient in terms of efficiency and satisfaction respectively [19]. Moreover, fibrin and cyanoacrylate glues decrease the exposure of foreign- bodies; therefore, it is especially useful in patients with needle phobia or impaired immune systems [20]. However, there are restrictions. The generalizability of the results is limited by a rather small size of the sample and a 3-month follow-up interval. Future development of adhesive biomaterials to optimize periodontal usage should have been supported by histological studies and enhance the further optimistic randomized controlled trials to prove the current research results.

## Conclusion:

Fibrin sealant (Tisseel) and cyanoacrylate adhesive (Amcrylate) were found to be better in terms of early wound healing, deflation of postoperative pain and excellent tissue biocompatibility. The two adhesives recorded full healing in one month and similar clinical results in three months. Tissue adhesives are therefore a consistent, secure and effectively efficient alternative to periodontal surgery flap closure.

## Figures and Tables

**Table 1:** Mean landry healing index

**Duration**	**Silk**	**Vicryl**	**Tisseel**	**Amcrylate**	**p-value**
Day 8	2.40 ± 0.52	3.50 ± 0.53	4.00 ± 0.67	3.70 ± 0.48	0
Day 15	3.70 ± 0.48	4.70 ± 0.48	4.80 ± 0.42	4.30 ± 0.48	0
Day 30	4.60 ± 0.52	5.00 ± 0.00	5.00 ± 0.00	5.00 ± 0.00	0.01
3 months	5.00 ± 0.00	5.00 ± 0.00	5.00 ± 0.00	5.00 ± 0.00	1

**Table 2:** Mean VAS Scores

**Duration**	**Silk**	**Vicryl**	**Tisseel**	**Amcrylate**	**p-value**
Day 8	5.20 ± 1.03	2.20 ± 0.63	0.60 ± 0.52	1.30 ± 0.48	0
Day 15	2.70 ± 0.95	0.40 ± 0.52	0.00 ± 0.00	1.20 ± 0.42	0

**Table 3:** Mean plaque and gingival indices

**Duration**	**Silk**	**Vicryl**	**Tisseel**	**Amcrylate**
Baseline	0.60 ± 0.52	0.40 ± 0.52	0.50 ± 0.53	0.60 ± 0.52
Day 8	1.50 ± 0.53	0.30 ± 0.48	0.30 ± 0.48	0.50 ± 0.53
Day 15	0.20 ± 0.42	0.30 ± 0.48	0.00 ± 0.00	0.00 ± 0.00

**Table 4:** Mean PPD and CAL Values

**Time**	**PPD (mm)**	**p-value**	**CAL (mm)**	**p-value**
Baseline	6.50 ± 2.10	0.84	6.70 ± 2.21	0.84
1 month	5.30 ± 0.80	0.56	5.10 ± 0.74	0.56
3 months	3.80 ± 0.70	0.1	3.70 ± 0.67	0.1
